# Late attentional processes potentially compensate for early perceptual multisensory integration deficits in children with autism: evidence from evoked potentials

**DOI:** 10.1038/s41598-020-73022-2

**Published:** 2020-09-30

**Authors:** Maria Elena Stefanou, Neil M. Dundon, Patricia E. G. Bestelmeyer, Chara Ioannou, Stephan Bender, Monica Biscaldi, Nikolaos Smyrnis, Christoph Klein

**Affiliations:** 1 Department of Child and Adolescent Psychiatry, Psychotherapy, and Psychosomatics, Medical Faculty, University of Freiburg, Hauptstrasse 8, 79104 Freiburg, Germany; 2 School of Psychology and Clinical Language Sciences, University of Reading, Reading, RG6 6AL UK; 3 Brain Imaging Center, Department of Psychological and Brain Sciences, University of California, Santa Barbara, CA USA; 4 School of Psychology, Bangor University, Bangor University, Bangor, LL57 2AS Gwynedd UK; 5 Department of Child and Adolescent Psychiatry, Medical Faculty, University of Cologne, 50931 Cologne, Germany; 6 Department of Psychiatry, Medical School, National and Kapodistrian University of Athens, Eginition Hospital, 11528 Athens, Greece

**Keywords:** Cognitive neuroscience, Autism spectrum disorders

## Abstract

Sensory processing deficits and altered long-range connectivity putatively underlie Multisensory Integration (MSI) deficits in Autism Spectrum Disorder (ASD). The present study set out to investigate non-social MSI stimuli and their electrophysiological correlates in young neurotypical adolescents and adolescents with ASD. We report robust MSI effects at behavioural and electrophysiological levels. Both groups demonstrated normal behavioural MSI. However, at the neurophysiological level, the ASD group showed less MSI-related reduction of the visual P100 latency, greater MSI-related slowing of the auditory P200 and an overall temporally delayed and spatially constrained onset of MSI. Given the task design and patient sample, and the age of our participants, we argue that electro-cortical indices of MSI deficits in ASD: (a) can be detected in early-adolescent ASD, (b) occur at early stages of perceptual processing, (c) can possibly be compensated by later attentional processes, (d) thus leading to normal MSI at the behavioural level.

## Introduction

Autism Spectrum Disorder (ASD) is a neurodevelopmental disorder characterised by qualitative impairments in social interaction and communication, and restricted, repetitive and stereotypic patterns of behaviour. Previously unreported features of ASD now featuring in the DSM-5^1^ are the hyper- or hypo-reactivity to sensory information and unusual interests in sensory aspects of the environment. Accordingly, empirical studies demonstrate that autistic individuals exhibit altered sensory processing in several domains, such as sensation seeking/sensitivity, low registration and avoidance^2^, and hyper-reactivity to acoustic stimuli^3,4^. Increased self-rated sensory responsivity also correlates positively with autistic traits in both autistic and neurotypical adults^5^.

Another implication of the sensory symptoms characteristic to autism are deficits in multisensory integration (MSI). MSI describes the combination of information about a single event arriving through multiple sensory channels either in temporal congruence, or at least occurring within a narrow temporal binding window^6^. MSI causes multimodal information to be processed faster and more accurately than unimodal information. MSI facilitates detection, for example, when aligning sound to a visual target, but can also elicit multisensory illusions such as when multiple adjacent tones alter the perceived number of flashes^7–10^. MSI-related effects are clearly observed in healthy children as early as 7 years old and seem to appear from the age of four and improve thereafter^7,11^. That is, behavioural responses improve while maturation of MSI processes is seen at the physiological level, e.g. as amplitude increase of the auditory N100^7^. Electrophysiological studies reveal that bimodal stimuli modulate the latency and amplitude of sensory-specific event-related potentials (ERPs) such as increased positivity for the visual P100^12^ and auditory P200^13^, increased negativity for the visual and auditory N1^7^, and reduced latency for the face-specific N170^14^. MSI also drives non-linear bimodal interactions at various temporal stages of processing, i.e., electrophysiological activity in sensory relevant areas in bimodal conditions exceeds activity predicted by summing the two composite unimodal conditions, an effect observed both in adult groups^13,15,16^ and children^17^.

Autistic individuals are impaired in a variety of MSI tasks, including the pip-and-pop visual search task, audio-visual gap/overlap tasks, simple reaction time paradigms, and two-choice discrimination tasks employing emotions^8,18,19^. Relative to controls, they benefit less from bimodal stimuli in terms of accuracy^8^ and reaction times (RTs)^8,18,19^, and show decreased sensitivity to multisensory illusions, probably indicating diminished MSI^9,10^. Importantly, it has been suggested that *a*-typicalities in the development of intersensory processing skills (perceiving unified information across modalities) would result in atypical social skills since several functions, including perception and social interaction, rely on the integration of constantly-changing information^20^. Thus, sensory and multisensory deficits could potentially lead to the social skills deficits seen in ASD, a fact supported by findings of a relation between audio-visual integration skills and deficits in communication and social skills^21,22^.

Amongst the studies which looked at MSI with EEG using non-social stimuli in children with ASD, an unexpected role of attention has emerged depending on task demands. By comparing the bimodally evoked potentials with the unimodal sum, studies^23^ reported a delayed MSI effect at about 300 ms, limited to parietal, parieto-occipital and centro-parietal areas, for children and adolescents with ASD compared to controls. Neurotypical individuals (henceforth called controls), in contrast, showed significant MSI activity as early as 120 ms evolving at frontal, central, parietal and central-parietal areas lasting until 200 ms. Notably, studies reported MSI effects with a widespread topography including frontal, fronto-central and occipital areas^18^. This effects were as early as 40ms^15,18^ sugegsting that the integration of the bimodal stimuli generated activity in the visual cortex in neurons not dedicated only to visual signals^15^. However, autistic individuals showed reduced electrophysiological MSI effects that were topographically restricted (post-hoc analysis revealed no latency differences)^18^. The different latency of the first observable significant MSI effects between the two aforementioned studies^18,23^ might result^18^ from different attention demands between the employed tasks. In the task showing delayed neural MSI responses^19^, participants were instructed to ignore the stimuli, while the task showing no latency effect required participants to actively attend to stimuli in order to respond^18^. This suggests that autistic individuals possibly need active attentional tone in order to initiate “early” perceptual MSI processes, which would otherwise be elicited spontaneously in healthy controls^18^. Indeed, it has been suggested that attention can affect MSI both in controls^24^ and autistic individuals^25^.

Studies^26^ further reported that a smaller auditory N1, a larger N1b and larger MSI neural responses 100-130 ms post-stimulus onset correlated with less severity of autistic symptoms in children and adolescents with ASD. According to the authors^26^, the N1 correlation with symptom severity could relate with the auditory cortex pathology reported in ASD while the MSI correlations suggest that MSI deficits are associated with the core symptomatology of the disorder (thus, leading to social deficits as well).

Based on these considerations, we aimed to investigate the electrophysiological correlates of MSI and its spatio-temporal evolution in autistic individuals between the ages of 11 and 14 years old, and *without ADHD comorbidity*, using a modified version of previously employed MSI paradigm to assay the modulatory role of attention.

We first assessed the effect of the redundant signal in the RTs of the bimodal vs unimodal conditions. We applied Miller’s Race Model Inequality (RMI)^27^ to verify whether a speed-up of RTs during the bimodal condition was due to MSI or statistical facilitation; that is, a race between the two signals of the bimodal condition. Given the recent changes in DSM-5^1^ allowing a comorbid diagnosis of Attention Deficit/Hyperactivity Disorder (ADHD), that 30–80% of autistic individuals fulfil the ADHD diagnostic criteria^28^ and recent reports of altered temporal binding window in participants with high scores of ADHD symptomatology^29^, we recruited adolescents without ADHD comorbidity. Furthermore, since MSI improves throughout childhood and adolescence, we recruited participants in a narrow age-range to minimise within-group heterogeneity and the confounding effects of neurodevelopment on both behavioural and electrophysiological correlates of MSI.

*Firstly*, we hypothesised that bimodal stimuli would cause faster RTs and increased accuracy due to MSI. We expected MSI to facilitate the consistency of RTs (the standard deviation of RTs, RTSD)^26,27^. *Secondly*, we expected the MSI behavioural facilitation to be served by changes in sensory-specific ERPs, specifically, decreased latency and increased amplitude of visual components during the bimodal condition and decreased amplitude of auditory components during the bimodal condition. We expected these MSI effects to be stronger for controls than the ASD group. *Thirdly,* for the control group, we expected electrophysiological MSI effects (across time and regardless of sensory-specific components) to onset early after stimulus presentation, extending over a prolonged time course and several scalp regions. In contrast, we expected temporally delayed and spatially constrained MSI effects in the ASD group.

## Results

### Behavioural results

Participants performed speeded responses to either bimodal or unimodal stimuli. As expected, the bimodal condition produced faster and less variable responses (Condition effects for RTs: *F*_(2, 80)_ = 108.86, *p* < *0.0*01, η_p_^2^ = 0.73; for SDRT: *F*_(2, 80)_ = 59.25, *p* < *0.0*01, η_p_^2^ = 0.60; Table 1) compared to both unimodal conditions (all *p* < *0.0*01). Accuracy was also significantly increased (Condition: *F*_(2,80)_ = 19.13, *p* < *0.0*01, η_p_^2^ = 0.32; Table 1) during the bimodal compared to the auditory (*p* = 0.002) or visual (*p* < 0.001) conditions. Controls showed higher overall accuracy (Group: *F*_(1,40)_ = 5.70, *p* = 0.022, η_p_^2^ = 0.13; Table 1) compared to the ASD group, and a trend for smaller variability of responses (Group: *F*_(1,40)_ = 3.64, *p* = 0.064, η_p_^2^ = 0.08). After adjusting the group means for IQ, groups did not differ anymore in accuracy (Group: *F*_(1,40)_ = 1.23, *p* = 0.274).

**Table 1:** Behavioural Responses. The table shows the median RTs, accuracy and SDRTs for each group; Standard Error (SE) in parenthesis.

	Accuracy			Median RTs			SDRTs		
	Audio	Bimodal	Visual	Audio	Bimodal	Visual	Audio	Bimodal	Visual
TDs	99.3 (0.2)	99.9 (0.1)	98.8 (0.3)	411.5 (18.18)	367.9 (12.0)	453.2 (12.4)	128.8 (8.9)	85.2 (6.1)	104 (6.8)
ASD	98.8 (0.3)	99.6 (0.1)	98.0 (0.3)	443.5 (22.1)	385.2 (14.6)	470.6 (15.1)	148.3 (10.8)	101.5 (7.3)	128.3 (8.2)

In the bimodal condition, both groups showed substantial Redundancy Gain (RG), namely 14.58% ± 5.59% for the ASD group and 12.82% ± 6.79% for controls. One-way ANOVAs revealed that groups did not differ in RG. Overall, both groups showed robust MSI in terms of RTs and RG with no significant group differences.

In the bimodal condition, both groups showed substantial Redundancy Gain (RG), namely 14.58% ± 5.59% for the ASD group and 12.82% ± 6.79% for controls. One-way ANOVAs revealed that groups did not differ in RG. Overall, both groups showed robust MSI in terms of RTs and RG with no significant group differences.

### Miller’s race model inequality

RTs facilitation in bimodal conditions may be caused by non-linear multisensory signal integration, that is, speeded RTs due to coactivation by the two signals^27^. However, the speed-up of RTs can be alternatively explained by a continual race of the two signals, and given independent variances in both latency distributions, one signal may trigger the response first probabilistically resulting to a speed-up of RTs not caused by MSI^27^. We accordingly fitted Miller’s RMI, comparing bimodal time-bins against the bound, i.e., the fastest time possible from the above probabilistic “race” (see Methods). RMI was calculated for each participant and for valid RTs (≥ 150 ms). Percentiles with values above zero (percentiles of the bimodal distribution being faster than the bound) represent MSI. Percentiles from each participant’s distribution were submitted to a one-sample t-test analysis per group to examine whether MSI was significant.

**Figure 1:**
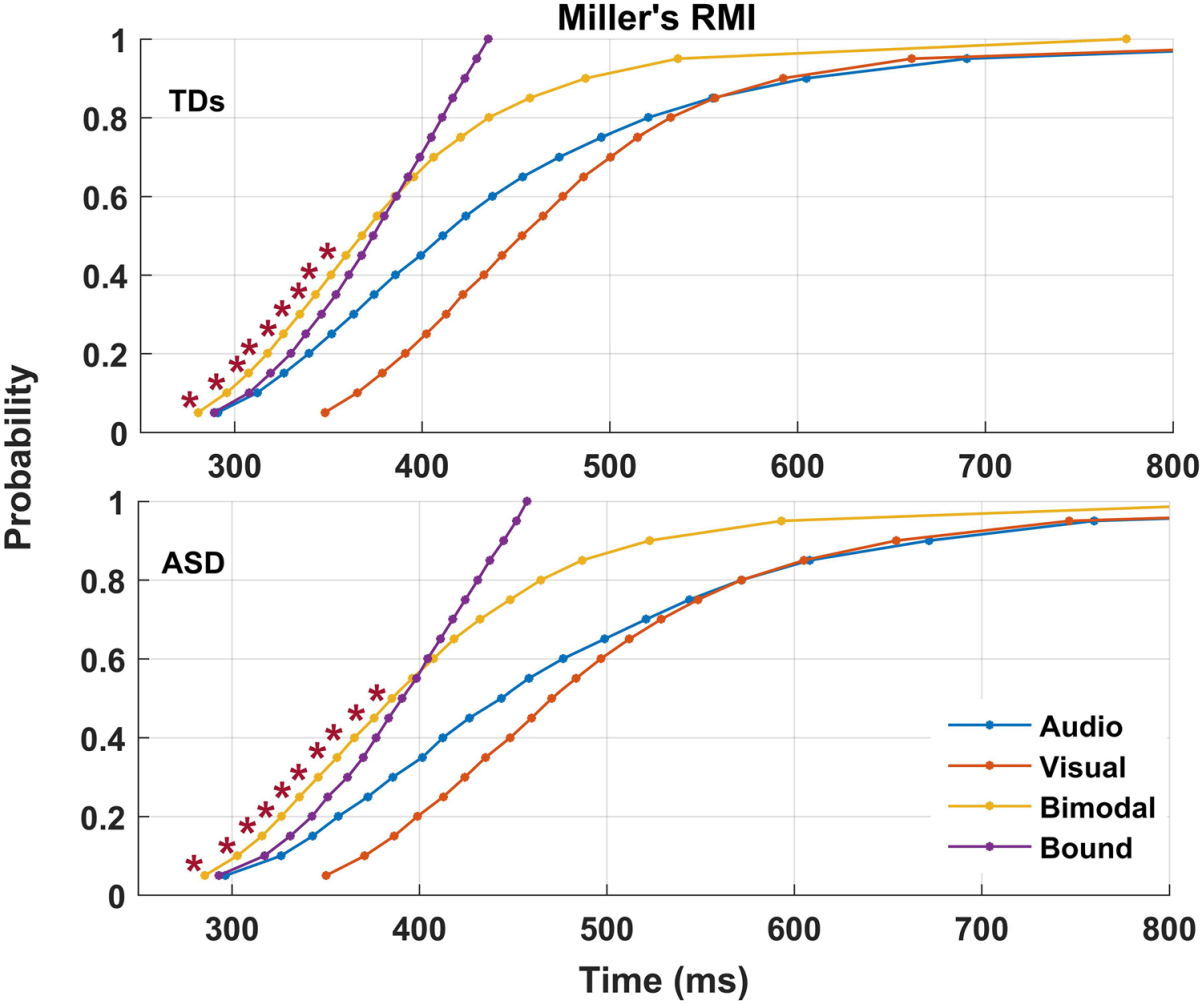
Miller’s race model inequality. The top figure illustrates the violation of Miller’s RMI for healthy children as seen by comparing the bimodal condition with the bound. The lower figure illustrates the violation of Miller’s RM for the ASD group. Asterisks signify the percentiles of reaction time bins with significant violation of the model, and thus MSI.

Miller’s RMI confirmed a significant MSI during the earliest percentiles of the RTs distribution for both groups – from the 5th to 50th percentile for the ASD group and from the 4th to the 45th for controls (Fig. 1).

## EEG results

### Early sensory components

We entered the peak amplitudes and latencies of the visual P100, N1, auditory N100, P200, and Late Positive Component in ANOVAs. Given that our aim was to investigate the MSI effect on the visual and auditory stream we contrasted the bimodal condition with the corresponding unimodal. Furthermore, the visual components are expected in posterior-occipital areas and the auditory components at central areas. Due to volume conduction and mixing of signals, the creation of the sum of the two unimodal condition with the sole purpose of investigating a sensory-specific component, e.g. the visual P100, would distort this component. We instead assessed the effect of the redundant multisensory signal on the sensory-specific components. That is, the effect of the redundant auditory information on the visual processing as indexed by the visual ERPs and the effect of the redundant visual information on the auditory processing as indexed by the auditory ERPs. Following the steps described in Methods, 82% of the ASD group maintained 400 segments or more and 18% less than 400 segments (minimum number of segments = 345). Similarly, 88% of the control group maintained 400 segments or more and 12% less than 400 segments (minimum number of segments = 352).

#### Visual P100

**Figure 2:**
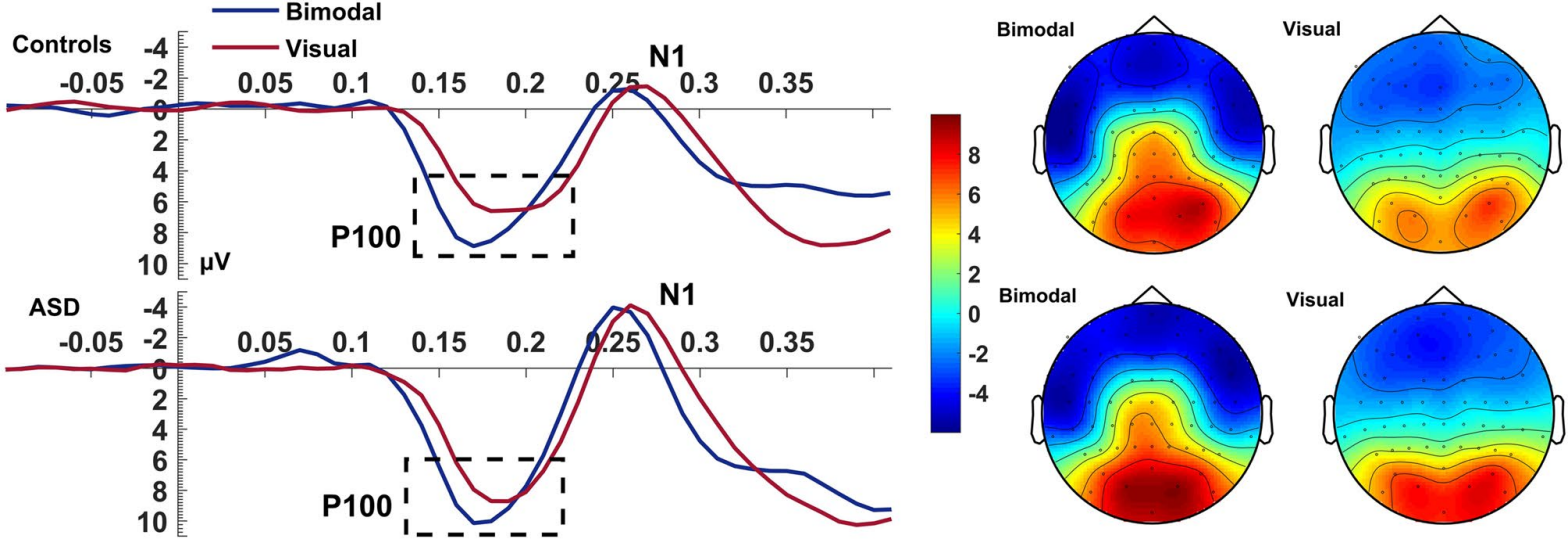
Visual P100. Average waveform (O1, O2) and topographies illustrating the visual P100 component at the bimodal and visual conditions, for controls and ASD. The dashed box highlights the amplitude increase during the bimodal condition compared to the visual, and the speeded latency of the component which is nearly double in controls than the ASD group. The Y-axis of the waveform represents activity in µV and the X-axis time in ms. The colour bar represents the range of activity in µV for interpretation of the topographical maps.

The bimodal condition produced an overall higher amplitude and a shorter latency compared to the visual condition (Condition effects on: amplitude: *F*_(1,40)_ = 13.72, *p* = 0.001, η_p_^2^ = 0.25; latency: *F*_(1,40)_ = 27.16, *p* < 0.001, η_p_^2^ = 0.40; Fig. 2). The latency reduction of P100 during the bimodal condition was, as seen in Fig. 2, almost twice as large in controls (Condition: *F*_(1,24)_ = 26.09, *p* < 0.001, η_p_^2^ = 0.52) than in autistic individuals (Condition: *F*_(1,16)_ = 6.51, *p* = 0.021, η_p_^2^ = 0.29) with the Group*Condition interaction of the main ANOVA exhibiting a trend (*F*_(1,40)_ = 3.46, *p* = 0.070, η_p_^2^ = 0.08). Despite this interaction remaining a trend, it corresponds to a medium effect size (*d* = 0.59). Combined with the almost double latency reduction in controls compared to autistic individuals, this interaction probably remained a trend due to our sample size.

#### Visual N1

**Figure 3:**
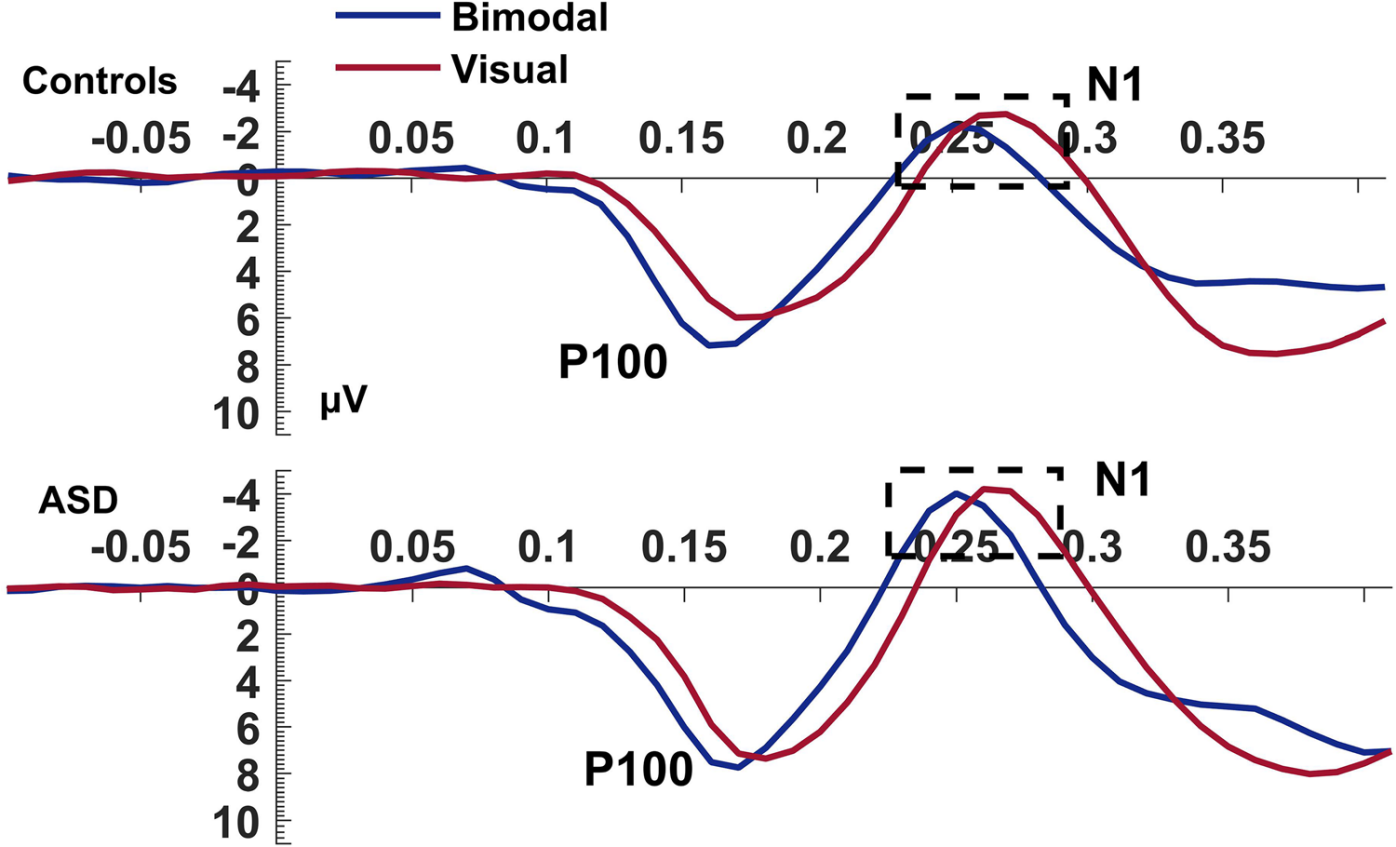
Visual N1. Average waveform (PO7, PO8, PO9, PO10) of the visual N1 component at the bimodal and visual conditions, for controls and ASD. The dashed box highlights the latency speedup of the component during the bimodal compared to the visual condition. The Y-axis of the waveform represents activity in µV and the X-axis time in ms.

The N1 component of the bimodal condition had a shorter latency compared to the visual condition (Condition: *F*_(1,40)_ = 29.27, *p* < 0.001, η_p_^2^ = 0.42; Fig. 3). No group differences were found.

#### Auditory N100

The auditory N100 component did not show any differences in amplitude or latency between the bimodal and auditory condition, nor did it differ between groups. The auditory N100 peaked earlier at electrode Cz compared to C1 (*p* = 0.003) but not compared to C2 (*p* > 0.05) (Electrode: *F*_(2,80)_ = 5.13, *p* = 0.008, η_p_^2^ = 0.11).

#### Auditory P200

**Figure 4:**
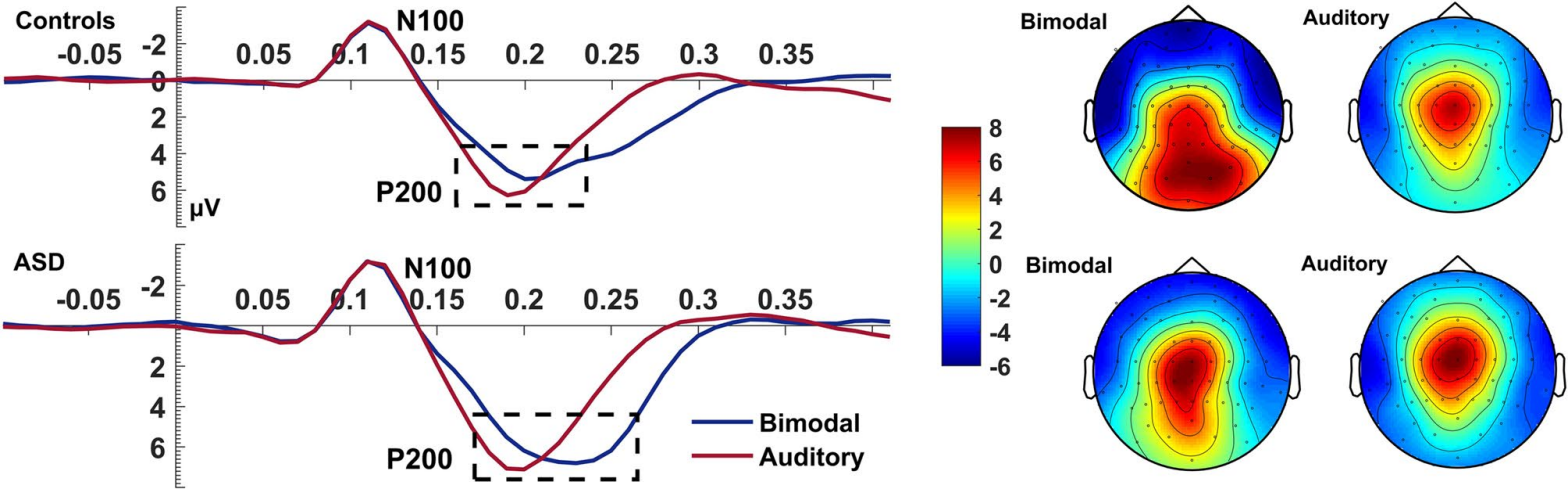
Auditory P200. Average waveform (C1, Cz, C2) and topographies of the auditory P200 component at the bimodal and auditory conditions, for controls and ASD. The dashed box highlights the latency delay of the component during the bimodal compared to the auditory condition which was increased more for the ASD group than for controls who also showed an overall speeded P200 compared to the ASD group. The Y-axis of the waveform represents activity in µV and the X-axis time in ms. The colour bar represents the range of activity in µV for interpretation of the topographical maps.

For the auditory P200, we found a significant Condition*Electrode*Group interaction (*F*_(2, 80)_ = 4.88, *p* = 0.010, η_p_^2^ = 0.11). Subsequent ANOVAs performed for each group separately revealed that P200 was delayed during the bimodal compared to the auditory condition and this effect was overall greater for the ASD group (CONDITION: *F*_(1,16)_ = 19.35, *p* < 0.001, η_p_^2^ = 0.55) than controls (Condition: *F*_(1,24)_ = 12.83, *p* = *0.002*, η_p_^2^ = 0.35; Fig. 4).

This delay of the bimodal compared to the auditory P200 in ASD was present regardless of electrode site whilst in controls, it differed between electrodes (Electrode: *F*_(2,48)_ = 3.89, *p* = 0.027, η_p_^2^ = 0.14; Condition*Electrode: *F*_(2,48)_ = 7.29, *p* = 0.002, η_p_^2^ = 0.23). This interaction seen in controls, whose P200 latency was overall faster than the ASD group (Group: *F*_(1,40)_ = 5.82, *p* = 0.021, η_p_^2^ = 0.13), was due to the delay of the bimodal compared to the auditory condition being significantly increased for Cz (*p* = 0.002) and C1 (*p* = 0.007) compared to C2.

P200 amplitude was highest at electrode Cz (Electrode: *F*_(2, 80)_ = 36.05, *p* < 0.001, η_p_^2^ = 0.48) compared to both C1 and C2 (*p* < 0.001). Electrode C1 also presented higher amplitude than C2 (*p* = 0.016). Finally, the redundancy of the bimodal condition did not affect the amplitude of P200, neither when directly contrasting the bimodal with the auditory condition (*p* = 0.728) nor through the difference waves (C1: *p* = 0.241; Cz: *p* = 0.934; C2: *p* = 0.210).

### Late positive component

There were no significant differences between conditions or groups in this component’s amplitude.

### IQ as a covariate

In order to examine whether our findings could be explained by the IQ difference between the two groups (Table 2), behavioural and ERP data were submitted to additional ANCOVAs with IQ as a covariate. As indicated above, IQ affected only the results of accuracy.

**Table 2:** Group characteristics and scores. *IQ is measured with the cultural fair intelligence test (CFT 20-Revised); **for the ASD group the N for SRS is N − 1, ADOS N − 3, ADOS II N = 3, and ADI-R is N − 1.

	TDs (N = 25)		ASD (N = 17)**			
	Mean (SD)	Range	Mean (SD)	Range	*t*	*p* values
Age	13.0 (0.90)	11.41–14.62	12.97 (0.96)	11.28–14.81	− 0.034	.973
IQ*	127.72 (16.2)	93–154	98.24 (17.92)	70–140	− 5.547	< .001
SRS raw	14.04 (15.92)	0–73	90.88 (29.14)	31–131	10.93	< .001
SRS T-norms	39.88 (13.15)	23–74	78.75 (10.81)	56–92	9.871	< .001
ADOS/ADOS II	N.A	N.A	12.71/8.67	6–22/2–16	–	–
ADI-R social interaction	N.A	N.A	15.94 (5.59)	3–25	–	–
ADI-R communication	N.A	N.A	11.06 (4.52)	3–18	–	–

### Spatio-temporal evolution of MSI

In order to investigate the spatio-temporal characteristics of MSI, we applied a non-parametric cluster-based permutation test. This analysis first accounts for the bimodal condition not simply comprising the sum of two unimodal signals^30^, but also a pure MSI component, which is not expected to manifest only during the latency of sensory components but rather early on after stimulus onset and throughout several latencies and scalp areas. Second, such non-parametric analysis copes with issues relating to multiple comparison problems when performing multiple t-tests across consecutive time points^23^. Third, it accounts for the dependency of the EEG data and it is free of assumptions with regards to the sample’s underlying distribution. In line with comparable studies^18^, amplitude changes between the two conditions were assessed from stimulus onset and until 300 ms post-stimulus onset, at every data point (i.e., every 10 ms) and across all channels.

**Figure 5:**
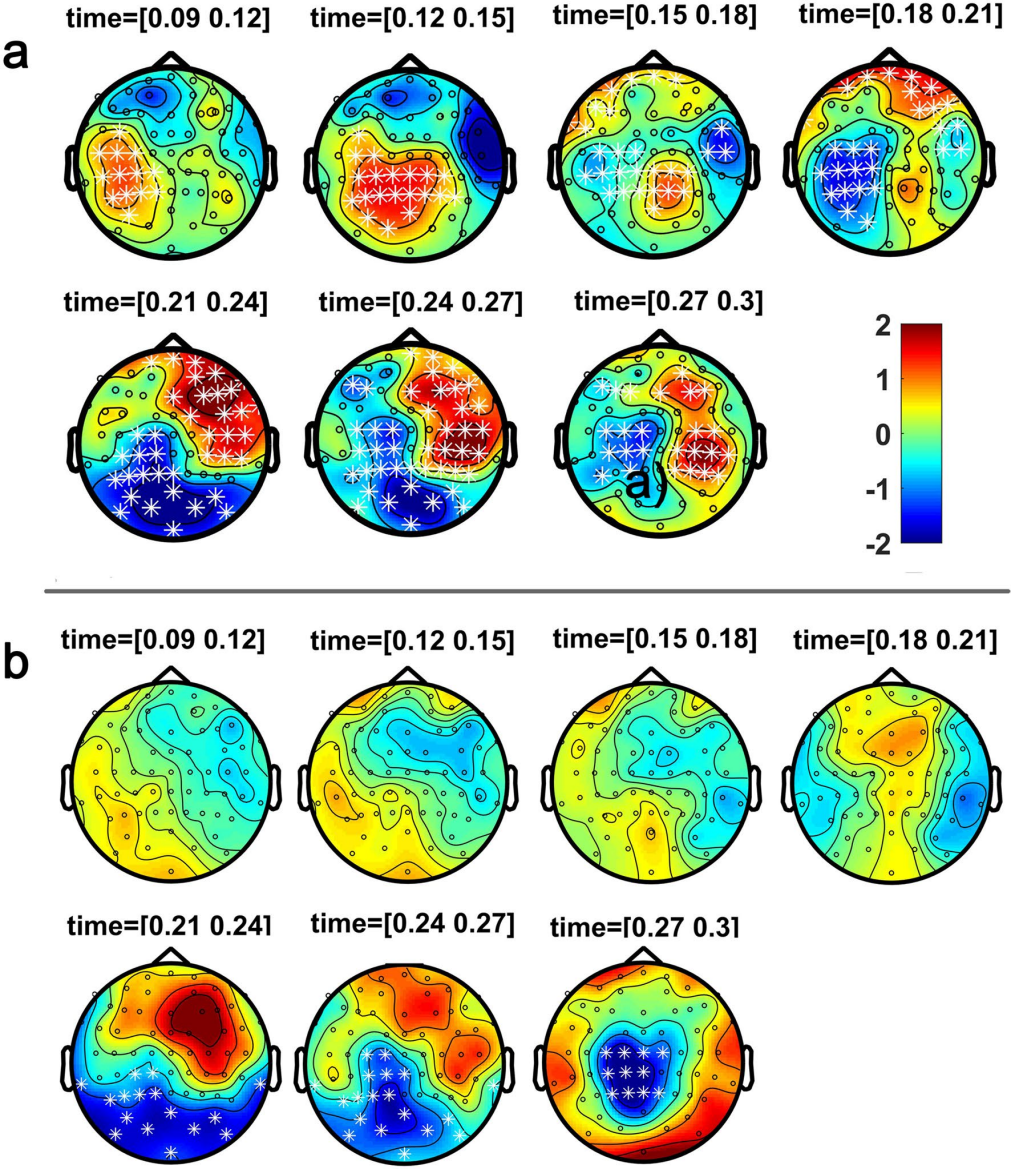
Spatio-temporal evolution of MSI. The figure illustrates the spatio-temporal course of the significant MSI effect [Bimodal – (Audio + Visual)] as seen through the cluster-based permutation test. Topographical maps of the clusters have been averaged in bins of 30 ms; white asterisks represent the significant effects for (**a**) controls, starting as early as 90 ms, and for (**b**) participants with ASD starting at 220 ms. The colour bar represents the range of activity in µV for interpretation of the topographical maps.

The cluster-based permutation test comparing bimodal and sum conditions, revealed significant MSI effects in controls as early as 90 ms and most pronounced at centro-temporal areas (*p* = 0.018) (Table 3 and Fig. 5). This MSI effect evolved to a more central topography up to 150 ms and from 170 ms up to 300 ms after stimulus onset with a widespread topographical distribution involving central and parieto-occipital (*p* = 0.002), and frontal and right fronto-temporal areas (*p* = 0.006). ASD participants also showed some MSI effects, beginning around 220 ms post-stimulus onset, i.e., 130 ms later than controls, and with a more restrained parieto-occipital distribution evolving to a constrained central topography from 250 ms onwards (*p* = 0.032; Fig. 5).

**Table 3:** Cluster-based permutation tests. The table shows the time-windows of clusters with significant differences between the bimodal and sum conditions for each group; Cluster statistic denotes the sum of the t-statistic for each cluster, df the degrees of freedom, *p* the significance value, SD the standard deviation and CI the Confidence Interval range.

	Cluster statistic (df)	*p*	SD	CI Range
**Controls**				
90–160 ms	260.58 (24)	.018	± 0.0042	± 0.0082
170–300 ms	433.40 (24)	.006	± 0.0024	± 0.0048
170–300 ms	− 456.89 (24)	.002	± 0.0014	± 0.0028
**ASD**				
220–300 ms	− 257.61 (16)	.032	± 0.0056	± 0.0109

## Discussion

The present study set out to investigate multisensory integration (MSI) in young adolescents with autism using simple visual and auditory stimuli that were either presented alone (unimodal conditions) or together (bimodal condition). We obtained the following main results. *First*, and regardless of group, we found robust behavioural MSI effects. Specifically, the bimodal condition produced overall faster and less variable RTs, in addition to increased accuracy compared to both unimodal conditions; bimodal stimuli also led to robust RG and significant MSI as defined by Miller’s RMI. However, ASD participants did not differ consistently from controls in the behavioural MSI effects, both in terms of RTs and as seen through Miller’s RMI. *Second*, MSI effects were readily observable in the sensory ERPs. That is, there was a faster visual P100 and N1 latency, and a delayed auditory P200 for the bimodal compared to the analogous unimodal conditions—the amplitude of the visual P100 was also increased during the bimodal compared to the visual condition. *Third*, and most notably, the electrophysiological results showed that autistic individuals exhibited reduced MSI-related latency reduction of the visual P100, an increased MSI-related delay of the auditory P200, and temporally delayed and spatially constrained electrophysiological MSI effects in a cluster-based permutation test.

In the present study, we replicated the robust behavioural effects of MSI in both the control and ASD groups. Like Brandwein et al.^7^ we found that MSI facilitation extends to simple audio-visual stimuli. The bimodal condition produced increased accuracy and faster RTs compared to the unimodal conditions, the latter of which was confirmed with Miller’s RMI as MSI.

The comparable behavioural results between the two groups is not entirely unprecedented. Several studies showed intact low-level MSI-driven RTs facilitation in ASD. Autistic individuals have been found to show MSI effects similar to controls in a pip-and-pop visual search task, as well as in MSI illusion paradigms^31–34^. Higher ASD susceptibility to the 2Flashes-1Beep illusion has also been reported (possibly due to an extended temporal binding window)^31^.

The lack of group behavioural differences could be driven by the addition of catch trials in order to increase attention demands. As previously shown^35^, catch trials encourage more “conservative” responses (slower saccadic RTs and less anticipatory responses) since participants need to withhold pre-potent responses. Thus, catch trials in our study could have produced “conservative” responses and increased attention. This effect would be stronger in the ASD group, as autistic individuals typically show reduced flexibility in set maintenance and shifting, both behaviourally^36,37^ and neurophysiologically^38^. Therefore, the increase in attention driven by catch trials might have been particularly pronounced in autistic individuals. Indeed, several studies have shown that attention improves MSI^24,25^. The discrepancies in MSI findings in ASD (studies showing intact MSI in ASD^31–34^)may suggest that autistic individuals are capable of integrating modalities as long as they actively attend to stimuli, while in controls, MSI facilitation arises spontaneously without the need for strong attentional tone^23^. This finding might bridge the conflicting findings reported previously. Brandwein et al.^18^ reported electrophysiological MSI effects in ASD, seen at ~ 100 ms postimulus; such an early MSI effect contrasts the delayed effects reported by others^23^, with the former authors remarking that this was possibly due to differences in attention demands between the tasks. By adapting Brandwein et al.’s^18^ procedures to include catch trials, we observe a pattern of results consistent with the Russo study^23^. Increased attention requirements in our study, relative to Brandwein et al.^18^, may explain the ASD group aligning their behavioural performance with controls. The increased attention possibly affected accuracy rates, which despite being statistically significant, are high enough (Table 1) to assume a ceiling effect.

Parallel pocessing frameworks of MSI argue that integration occurs at different stages, with MSI at early sensory and pre-attentive stages (early MSI) occurring automatically and independent of attention, while later MSI (late MSI) is necessarily modulated by attention^39^. MSI by top-down attention at later stages reinforces the hypothesis that autistic individuals can integrate as long as they attend, which might explain the lack of group differences at the behavioural level. Therefore, the increased attention could enable the ASD group to compensate for deficient early MSI at the late integration stage.

MSI effects were also seen through visual ERPs. In the bimodal condition, we observed increased amplitude of the visual P100 and faster P100 and N1 in both groups. The visual P100 and N1 are exogenous components elicited involuntarily at the appearance of a visual stimulus even when not attended, while the deflection of both components increases when a person allocates attention to the stimulus^40,41^. Although both components are elicited at stimulus detection and enlarged by attention, it has been suggested that the attentional effects on visual P100 and N1 represent different mechanisms^42,43^. Luck et al.^43^ showed that the P100 and N1 are distinct components, as P100 represents the facilitation of sensory processing for stimuli at attended locations while the N1 represents attentional orienting. More importantly, the visual P100 and N1 are distinct components not only functionally but the P100 and face-sensitive N170 (the visual N1 here) arise from independent neural networks^44^. In our study, the increased amplitude and faster peak latency of the bimodal visual P100, suggests a more robust and faster sensory processing due to the multimodality of the event. This amplitude increase is consistent with prior findings of super-additivity, that is, additional activity produced by MSI that leads to an increase in neurophysiological responses^7,30,45–47^. This super-additive effect of MSI is also in line with hypotheses derived from fMRI studies suggesting that brain areas involved in sensory-specific processing^48–50^ contribute to MSI. MSI also produced significantly faster latencies of the visual N1 in the bimodal compared to the visual condition, suggesting that MSI does not only facilitate perceptual processing but also accelerates attentional processes. Thus, MSI, along with the observed speeded processing in both groups, could have a role in fine-tuning the detection and processing of objects as soon as information has reached the visual cortex.

Given that the auditory N100 has been described as an initial orienting response^51^ and the P100 increases during attention allocation^40,41^, the absence of an MSI effect on the *auditory* N100 compared to the simultaneous *visual* P100 suggests that any early MSI modulation of sensory-specific areas is stronger for visual than auditory areas. This interpretation aligns with studies indicating the importance of the primary visual cortex as a region of MSI processes^49^.

While we observed no MSI effects on the auditory N100, we observed a delayed P200 in the bimodal compared to the auditory condition. The auditory P200 reflects sound encoding and discrimination and has been related to early attentive mechanisms^51^ and selective attention^52^. An increased P200 latency (i.e., slower) has been linked to improved discriminability^53^ and performance in selective attention tasks^54^. This specific finding may therefore point to increased sound discrimination in multimodal sensory events.

In contrast to the behavioural data, the electrophysiological analyses revealed significant differences between the ASD and control groups. The cluster-based permutation test showed that the earliest manifestation of MSI was seen earlier than basic sensory processing. Controls showed an MSI effect as early as 90 ms after stimulus onset at centro-temporal sites and up to 300 ms with a widespread topographical distribution including central-parietal, occipital and right lateralized fronto-temporal sites. Compared to controls, autistic individuals showed a later onset of the MSI effect at 220 ms post-stimulus onset and with a more constrained topography, shifting from parieto-occipital to central sites. These MSI effects are in line with studies^19^ reporting a substantial delay of any MSI effects in the ASD group compared to controls, and studies^18^ showing topographically widespread effects for controls but not autistic individuals. Although we do not replicate the same topographies and latencies, we report a delayed MSI effect in ASD suggesting a delayed and spatially constrained integrative process. This delayed integrative effect in ASD would be in line with parallel processing frameworks that distinguish between early and late MSI, with late MSI being modulated by attention^39^. Taken together, the early onset of MSI effects for controls, the delayed onset of MSI effects for austistic individuals and the similar topography between the two groups at 220–250 ms (before the motor response) provides further support that autistic individuals potentially compensate for any MSI deficits at later processing stages via top-down attention. The interpretation that attention is modulating a sensory deficit is also consistent with previous electrophysiological findings with different patient groups. Treatment-derived improvements in oculomotor function in hemianopia patients were previously demonstrated to coincide with modulations in ERP amplitudes in late (~ P300) ERP time windows^55^.

Shortly after the first evident manifestation of MSI in the electrophysiological data, we observed significantly faster visual P100 during the bimodal vs visual condition. This effect was twice as large in controls than in the ASD group. Given the P100′s role in processing stimuli at attended locations, its relatively reduced MSI-related latency facilitation in the ASD group is a further marker of deficient sensory processing that we observe in this group. However, at later components such as the visual N1, autistic individuals showed an MSI-related latency attenuation similar to that of controls. Since N1 reflects attentional orienting, the lack of group differences in N1 suggests that autistic individuals can show sufficient MSI once they orient and actively attend the multimodal object and present MSI deficits only at initial processing stages.

For the auditory P200, controls showed overall shorter latencies on unimodal auditory trials than the ASD group suggesting they process auditory information more efficiently. The MSI-related delay of the auditory P200 was also greater in participants with ASD than controls. Since *improved* performance and discriminability^54,56^ are linked to a *delayed* auditory P200 latency, the slower unimodal auditory P200 and greater MSI-related delay of this component in autistic individuals support our interpretation that a greater attentional effort is needed in ASD compared to controls in order to “catch-up” in auditory processing and “make up” for early MSI deficits in the bimodal condition. That autistic individuals showed the first integrative effects in the cluster-based permutation test at a latency that coincides with the P200 interval further supports our interpretation. This finding, combined with the increased attentional effort indexed by the P200 delay, argues that autistic individuals compensate for any early perceptual (i.e., P100) and MSI deficits by a later attentional effort, which can be assayed with sufficient attentional demands in MSI paradigms.

The present study provides evidence of robust MSI using a simple RTs task. The absence of group differences at the behavioural level contrasts with observed differences at the neurophysiological level. However, previous MRI and EEG studies have reported between—or within-subjects effects that were observed the a neurophysiological but not the behavioural level^46,57^. Neurophysiology may be more sensitive than behaviour in differentiating groups and/or conditions. In addition, there is support that autistic individuals show sufficient MSI when deploying appropriate levels of attention. Our electrophysiological findings reveal MSI processing in ASD with a delayed temporal course compared to controls, i.e., they show MSI deficits only at early sensory processing stages and compensate later. Furthermore, the cluster-based permutation analyses suggests that MSI effects in ASD occur later and in topographically more constrained cortical generator structures.

Impairments of long-range connectivity, which is the structural and functional connectivity between distal brain regions, have been implicated in ASD^50,58,59^. Studies have reported reduced functional connectivity between several regions including the visual cortex and the inferior frontal area in ASD^60^ and increased connectivity between thalamus and auditory, somatosensory cortices^61^. The delayed spatio-temporal MSI effect reported in the above studies and the extended temporal binding window reported in ASD^33^ would be in line with impaired long-range connectivity in ASD. Therefore, sensory processing deficits, along with the altered connectivity found in ASD^61–63^ and its suggested role in MSI deficits (via insufficient signalling and synchronisation between the involved areas^64,65^) have made this a topic of increased interest in ASD research during the past few years^64^.

In sum, the results of the present study reveal that under appropriate experimental conditions, early perceptual MSI deficit in ASD can be compensated for by later attentional processes and thus does not manifest at the behavioural level.

The present study has some limitations that narrow the generalisability of our findings. Firstly, the sample size was overall rather small. This reduced the statistical power of the study and required effect sizes discussions of some our findings. Secondly, the age range was rather limited, thus limiting the developmental implications of the MSI effects reported here. Thirdly, a substantial proportion of autistic individuals are comorbid for ADHD. The exclusion of such cases by design implies that our results have no bearing for this subgroup. Nonetheless, the current results add to the literature suggesting that autistic individuals can integrate information with increased attention to the task. Pending an independent replication of our results, the presented study suggests that at least some of the “low-level” perceptual anomalies may be compensateable by “high-level” top-down control.

## Methods

### Participants

A total of 50 children (21 with ASD; 29 controls) between the ages of 11 and 14 years were invited to participate in the study. All children were recruited through the database of the Clinic for Child and Adolescent Psychiatry, Psychotherapy, and Psychosomatics of the University of Freiburg. Both groups were administered the following questionnaires: Social Responsivity Scale^66^ (SRS; parental assessment of autistic traits); and External Assessment Form (completed by parents/legal guardians) and Self-Assessment Form for Attention Deficit / Hyperactivity both from the *DISYPS-II*^67^. In addition, we gathered the following socio-demographic questionnaire data: age, type of school, grades, usage of media, presence of medication or medical treatment (either by a general practitioner or by a psychiatrist/psychologist), sleep patterns, education and occupation of parents.

Participants of the ASD group had been diagnosed with Autism Spectrum Disorder (F84.0, F84.1, F84.5) by an experienced psychiatrist/psychologist in the clinic as per the International Classification of Diseases^68^ (ICD-10-R). Diagnoses were based on anamnestic interviews with parents and children, the administration of the German version of Autism Diagnostic Observation Schedules^69^ (ADOS) and the Autism Diagnostic Interview-Revised^70^ (ADI-R). ADOS-2^71^ was used with four participants. All participants with autism were medication-free with the exception of two autistic individuals. One participant not fulfilling the ADHD diagnostic criteria was given methylphenidate in an attempt to address irritability and inattention in social situations that could be explained clinically by the autistic core symptomatology. This participant was medication-free during the testing (paused treatment 24 h prior to the testing sessions). A second participant was receiving antipsychotics (Abilify) due to a comorbid diagnosis of obsessive–compulsive disorder. Exclusion criteria for both groups were a first language other than German, comorbid diagnoses such as motor tics, epilepsy, ADHD, or an IQ score below 70 (as assessed with the Cultural Fair Intelligence Test 20-R, CFT 20-R^72^).

Furthermore, participants were excluded from data analysis if EEG data were heavily contaminated by muscle or movement artefacts, and in cases where they failed or refused to complete at least five out of 6 blocks per task. For healthy children, scores outside the normal range for the SRS was also an exclusion criterion.

After the application of the above criteria, our sample decreased to 17 children with ASD (11 male, 16 right-handed) and 25 healthy children (13 male, 22 right-handed; Table 2). Participants were compensated for their time with one cinema or book voucher (worth 7.50 €) per hour.

The experimental protocol was approved by the Ethics Committee of the Albert Ludwigs-University of Freiburg, and all data were treated in accordance with the declaration of Helsinki. Participants and their parents/legal guardians provided informed written consent, after a verbal and written description of the study. The study was conducted in the departmental EEG laboratory.

### Stimuli and procedure

The experiment was part of a larger study^73^ and was completed over three sessions. In the first two sessions, participants completed two MSI tasks with simultaneously recorded EEG. The third appointment was dedicated to the administration of the Cultural Fair Intelligence Test 20-R^72^ (CFT 20-R).

Participants were seated inside a dimly lit Faraday cage at an approximate viewing distance of 90 cm from the viewing monitor (61 cm diagonal, 60 Hz refresh rate). Experimental stimuli were presented using Psychophysics Toolbox extensions 3.0.12^74–76^ on MATLAB R2015a (The MathWorks, Inc., Natick, Massachusetts, United States).

The MSI task was guided by the procedures of Brandwein et al.^18^, and required participants to respond as quickly and as accurately as possible to one of three targets: auditory, visual and bimodal auditory + visual. Notably, Gondan and Minakata^77^ have recently explained how anticipatory responses lead in guessing bias and an unreliable estimation of the RTs distribution when testing MSI and Miller’s RMI. They suggest the use of a pre-stimulus interval pooled from an exponential distribution and the addition of catch trials to decrease such biases^77^. Furthermore, it has been suggested^18^ that attention allocation could affect, at a first stage at least, the electrophysiological MSI effects seen in participants with ASD and we thus aimed to explore to which extent this holds true. For these reasons, both the said variation of pre-stimulus intervals and the addition of catch trials were implemented. A new trial was indicated by a blank screen (black background) of 200 ms, followed by a white fixation cross, which remained at the centre of the screen for the rest of the trial. After a pre-stimulus interval of 2000–3000 ms (taken from an exponential distribution with a mean of 2400 ms), stimuli appeared for 60 ms; on visual trials, a red disc (diameter 1.5°) located 1° above the screen’s centre, and on auditory trials, a 1000 Hz tone, delivered through speakers located behind the screen. The bimodal condition included the simultaneous presentation of the disc and the tone. During catch trials, only the fixation cross was presented. Participants had to press key “1” with the index finger of their right hand every time a stimulus appeared and withhold responses during catch trials, within a post-stimulus response window of 1400 ms. Participants performed a total of 6 blocks of 100 trials, totalling 150 trials per experimental condition and 150 catch trials.

Brain Vision Recorder (Brain Products, Gilching), two BrainAmps DC amplifiers and a 64-channel actiCap (Brain Products, Gilching) were used for the acquisition of EEG according to the International 10–10 System^78^. The EEG was recorded with a 500 Hz sampling rate, with impedances kept below 5kΩ. FCz and AFz electrodes served as reference and ground, respectively. Finally, two infraorbital channels were placed vertically under each eye, and an additional electrode was positioned at the Nasion.

### Data processing and analysis

#### Behavioural data

Valid trials were defined as trials with correct responses ≥ 150 ms for the three main conditions and as the absence of a response for catch trials. Median reaction times (RTs), SDRT and the percentage of correct responses were submitted to a 2*3 mixed model ANOVA with Group (ASD, controls) as between-subjects factor and Condition (auditory, visual, bimodal) as within-subjects factor. Catch trials were not analysed (lowest accuracy of catch trials was at 91%).

Redundancy gain (RG) was calculated as the decrease of RTs (in percentage) during the bimodal compared to the fastest unimodal condition for each participant in each group. RG was submitted to a one-way ANOVA to compare groups. Furthermore, we applied Miller’s Race Model Inequality^27^ (RMI; as described in Ulrich, Miller & Schröter^79^). RTs distributions for the auditory, visual, bimodal and the bound (hypothetical bimodal) were calculated at every 5th percentile of the distribution (5th–100th percentile).

#### EEG data

EEG data were processed offline with Brain Vision Analyzer (Version 2.0, Brain Products, Gilching). Firstly, data were down-sampled to 100 Hz, and a 0.1–40 Hz bandpass filter was applied. Secondly, data sections with a voltage of ≤ 0.5 μV or ≥ 1500 μV and duration of ≥ 200 ms were considered as artefact-contaminated and were excluded from further analysis (including data ± 200 ms relative to the artefact). Thirdly, data were segmented into epochs beginning 200 ms prior to and ending 1500 ms after stimulus onset, resulting in 1700 ms epochs. Segments were then submitted to an Infomax Independent Component Analysis (ICA) and all components representing artefacts such as eye blinks, saccades, muscle activity and other movements were removed and not back-projected to the electrode space through a semi-automatic ICA Inverse. An additional data inspection was performed and any trials with activity ≤ 0.5 μV or ≥ 200 μV for a period ≥ 200 ms were again excluded. This data inspection was performed in a semi-automatic mode in order to also visually inspect the selected trials. Data were then re-referenced to the average reference, data of both sessions were appended and segments were averaged according to condition. Individual averaged ERPs were created after baseline was normalised to the 200 ms pre-stimulus period.

##### Traditional ERPs

The sensory-specific components were identified based on the latency and topography of the corresponding component. That is, we identified the visual P100 and N1 at the expected occipital and parietal-occipital areas^80^ and the auditory N100 and P200 at central areas^52^. Time-windows for peak amplitudes and latencies were chosen after visual inspection of grand averages as well as of individual averages in order to account for possible inter-individual variance of peak latencies. Peaks were identified within the following time windows: 70–140 ms for the auditory N100; 170–240 ms for the auditory P200; 80–220 ms for the visual P100; and 160–270 ms for the visual N1. Furthermore, a late positive component was identified in all three conditions. The component identified in the auditory condition peaked at 290 ms and was most prominent at electrodes T8 and TP8, while in the visual condition the maximum amplitude was identified at electrodes PO7 and O1 at 380 ms. The peak picking procedure was done in a semi-automatic mode in order to visually verify the identified peaks. Peak latencies within relevant windows were exported, in addition to amplitudes values computed as the average activity ± 10 ms relative to a given peak.

All of the subsequent ANOVAs included Group (adolescents with ASD vs controls) as between-subjects factor. Amplitudes and latencies of the visual P100 were submitted to a 2*2*2 mixed model ANOVA with Condition (visual, bimodal) and Electrode (O1, O2) as within-subjects factors. For the visual N1, amplitudes and latencies were submitted to a 2*4*2 mixed model ANOVA with Condition (visual, bimodal) and Electrode (PO7, PO8, PO9, PO10) as within-subjects factors. With regards to the auditory N100 and P200, amplitudes and latencies were submitted to two separate 2*2*3 mixed-model ANOVAs with Condition (auditory, bimodal) and Electrode (C1, Cz, C2) as within-subjects factors.

For the late positive component, we ran two additional 2*2*2 mixed-model ANOVAs with Condition (visual/auditory, bimodal) and Electrode (PO7, O1 / T8, TP8) as within-subject factors. Due to the broad shape of this component, latencies were not analysed.

To compensate for sphericity violations in the ANOVAs conducted on behavioural and electrophysiological data, we report, were appropriate, the Greenhouse–Geisser corrected p-values along with the original degrees of freedom.

##### Spatio-temporal evolution of MSI

We also investigated the spatio-temporal course of MSI by contrasting the bimodal and sum condition, in each group separately. This assessment was statistically implemented in the Fieldtrip toolbox^81,82^ through a cluster-based permutation test using the non-parametric Monte Carlo method.

Since this analysis assumes the null hypothesis of no differences between conditions, the data from the two distributions (i.e., conditions) are exchangeable. Therefore, participants’ data (bimodal and sum conditions) were combined in one dataset, which was then randomly split into two partitions and were compared with t-tests for each group separately. The randomisation followed by the t-testing was repeated 10,000 times, thus creating a reference distribution for the comparison of our two conditions. Since this is a cluster permutation test, adjacent electrodes that show the same effect were clustered, the sum of the t-values within a cluster was used as the cluster-statistic and the cluster with the maximum sum was used as the test statistic.

## Data Availability

The datasets generated during and/or analysed during the current study are available from the corresponding author on reasonable request.

## References

[1] American Psychiatric Association. *Diagnostic and statistical manual of mental disorders*. (American Psychiatric Assiociation, 2013).

[2] Kern JK (2007). Examining sensory quadrants in autism. Res. Autism Spectr. Disord..

[3] Gomot M, Belmonte MK, Bullmore ET, Bernard FA, Baron-Cohen S (2008). Brain hyper-reactivity to auditory novel targets in children with high-functioning autism. Brain.

[4] Kujala T, Lepistö T, Näätänen R (2013). The neural basis of aberrant speech and audition in autism spectrum disorders. Neurosci. Biobehav. Rev..

[5] Tavassoli T, Miller LJ, Schoen SA, Nielsen DM, Baron-Cohen S (2014). Sensory over-responsivity in adults with autism spectrum conditions. Autism.

[6] Pöppel E (1997). A hierarchical model of temporal perception. Trends Cognit. Sci..

[7] Brandwein AB (2011). The development of audiovisual multisensory integration across childhood and early adolescence: a high-density electrical mapping study. Cereb. Cortex.

[8] Collignon O (2013). Reduced multisensory facilitation in persons with autism. Cortex.

[9] de Boer-Schellekens L, Keetels M, Eussen M, Vroomen J (2013). No evidence for impaired multisensory integration of low-level audiovisual stimuli in adolescents and young adults with autism spectrum disorders. Neuropsychologia.

[10] Stevenson RA (2014). Evidence for diminished multisensory integration in autism spectrum disorders. J. Autism Dev. Disord..

[11] Bair WN, Kiemel T, Jeka JJ, Clark JE (2007). Development of multisensory reweighting for posture control in children. Exp. Brain Res..

[12] Stefanou ME (2019). Electro-cortical correlates of multisensory integration using ecologically valid emotional stimuli: differential effects for fear and disgust. Biol. Psychol..

[13] Jessen S, Kotz SA (2011). The temporal dynamics of processing emotions from vocal, facial, and bodily expressions. Neuroimage.

[14] Brefczynski-Lewis J, Lowitszch S, Parsons M, Lemieux S, Puce A (2009). Audiovisual non-verbal dynamic faces elicit converging fMRI and ERP responses. Brain Topogr..

[15] Giard M, Peronnet F (1999). Auditory-visual integration during multimodal object recognition in humans: a behavioral and electrophysiological study. J. Cognit. Neurosci..

[16] Molholm S (2002). Multisensory auditory-visual interactions during early sensory processing in humans: a high-density electrical mapping study. Brain Res. Cognit. Brain Res..

[17] Brett-green BA, Miller LJ, Gavin WJ, Davies PL (2008). Multisensory integration in children: a preliminary ERP study. Brain Res..

[18] Brandwein AB (2013). The development of multisensory integration in high-functioning autism: High-density electrical mapping and psychophysical measures reveal impairments in the processing of audiovisual inputs. Cereb. Cortex.

[19] Charbonneau G (2013). Multilevel alterations in the processing of audio–visual emotion expressions in autism spectrum disorders. Neuropsychologia.

[20] Bahrick L, Todd J, Stein BE (2012). Multisensory processing in autism spectrum disorders: intersensory processing disturbance as a basis for atypical development. The New Handbook of Multisensory Processes.

[21] Mongillo EA (2008). Audiovisual processing in children with and without autism spectrum disorders. J. Autism Dev. Disord..

[22] Woynaroski TG (2013). Multisensory speech perception in children with autism spectrum disorders. J. Autism Dev. Disord..

[23] Russo N (2010). Multisensory processing in children with autism: High-density electrical mapping of auditory-somatosensory integration. Autism Res..

[24] Morís Fernández L, Visser M, Ventura-Campos N, Ávila C, Soto-Faraco S (2015). Top-down attention regulates the neural expression of audiovisual integration. Neuroimage.

[25] Magnée MJCM, de Gelder B, van Engeland H, Kemner C (2011). Multisensory integration and attention in autism spectrum disorder: evidence from event-related potentials. PLoS ONE.

[26] Brandwein AB (2015). Neurophysiological indices of atypical auditory processing and multisensory integration are associated with symptom severity in autism. J. Autism Dev. Disord..

[27] Miller J (1982). Divided attention: evidence for coactivation with redundant signals. Cognit. Psychol..

[28] Rommelse NNJ, Franke B, Geurts HM, Hartman CA, Buitelaar JK (2010). Shared heritability of attention-deficit/hyperactivity disorder and autism spectrum disorder. Eur. Child Adolesc. Psychiatry.

[29] Panagiotidi M, Overton PG, Stafford T (2017). Multisensory integration and ADHD-like traits: evidence for an abnormal temporal integration window in ADHD. Acta Psychol. (Amst).

[30] Stein BE, Stanford TR (2008). Multisensory integration: current issues from the perspective of the single neuron. Nat. Rev. Neurosci..

[31] Bao VA, Doobay V, Mottron L, Collignon O, Bertone A (2017). Multisensory integration of low-level information in autism spectrum disorder: measuring susceptibility to the flash-beep illusion. J. Autism Dev. Disord..

[32] Van Der Smagt MJ, Van Engeland H, Kemner C (2007). Brief report: can you see what is not there? Low-level auditory-visual integration in autism spectrum disorder. J. Autism Dev. Disord..

[33] Foss-Feig JH (2010). An extended multisensory temporal binding window in autism spectrum disorders. Exp. Brain Res..

[34] Kwakye LD, Foss-Feig JH, Cascio CJ, Stone WL, Wallace MT (2011). Altered auditory and multisensory temporal processing in autism spectrum disorders. Front. Integr. Neurosci..

[35] Kingstone A, Klein RM (1993). What are human express saccades?. Percept. Psychophys..

[36] Miller HL, Ragozzino ME, Cook EH, Sweeney JA, Mosconi MW (2015). Cognitive set shifting deficits and their relationship to repetitive behaviors in autism spectrum disorder. J. Autism Dev. Disord..

[37] Yerys BE (2009). Set-shifting in children with autism spectrum disorders. Autism.

[38] Doesburg SM, Vidal J, Taylor MJ (2013). Reduced theta connectivity during set-shifting in children with autism. Front. Hum. Neurosci..

[39] Koelewijn T, Bronkhorst A, Theeuwes J (2010). Attention and the multiple stages of multisensory integration: a review of audiovisual studies. Acta Psychol. (Amst).

[40] Mangun GR (1995). Neural mechanisms of visual selective attention. Psychophysiology.

[41] Hillyard SA, Vogel EK, Luck SJ (1998). Sensory gain control (amplification) as a mechanism of selective attention: electrophysiological and neuroimaging evidence. Philos. Trans. R. Soc. Lond. Ser. B. Biol. Sci..

[42] Heinze HJ, Luck SJ, Mangun GR, Hillyard SA (1990). Visual event-related potentials index focused attention within bilateral stimulus arrays. I. Evidence for early selection. Electroencephalogr. Clin. Neurophysiol..

[43] Luck SJ, Heinze HJ, Mangun GR, Hillyard SA (1990). Visual event-related potentials index focused attention within bilateral stimulus arrays. II. Functional dissociation of P1 and N1 components. Electroencephalogr. Clin. Neurophysiol..

[44] Desjardins JA, Segalowitz SJ (2013). Deconstructing the early visual electrocortical responses to face and house stimuli. J. Vis..

[45] Meredith M, Stein B (1983). Interactions among converging sensory inputs in the superior colliculus. Science.

[46] Santangelo V, Van Der Lubbe RHJ, Olivetti Belardinelli M, Postma A (2008). Multisensory integration affects ERP components elicited by exogenous cues. Exp. Brain Res..

[47] Senkowski D, Saint-Amour D, Höfle M, Foxe JJ (2011). Multisensory interactions in early evoked brain activity follow the principle of inverse effectiveness. Neuroimage.

[48] Macaluso E, Driver J (2005). Multisensory spatial interactions: a window onto functional integration in the human brain. Trends Neurosci..

[49] Murray MM (2016). The multisensory function of the human primary visual cortex. Neuropsychologia.

[50] Hornix BE, Havekes R, Kas MJH (2019). Multisensory cortical processing and dysfunction across the neuropsychiatric spectrum. Neurosci. Biobehav. Rev..

[51] O’Connor K (2012). Auditory processing in autism spectrum disorder: a review. Neurosci. Biobehav. Rev..

[52] Crowley KE, Colrain IM (2004). A review of the evidence for P2 being an independent component process: Age, sleep and modality. Clin. Neurophysiol..

[53] Lijffijt M (2009). P50, N100, and P200 sensory gating: Relationships with behavioral inhibition, attention, and working memory. Psychophysiology.

[54] Rif J, Hari R, Hämäläinen MS, Sams M (1991). Auditory attention affects two different areas in the human supratemporal cortex. Electroencephalogr. Clin. Neurophysiol..

[55] Dundon NM, Làdavas E, Maier ME, Bertini C (2015). Multisensory stimulation in hemianopic patients boosts orienting responses to the hemianopic field and reduces attentional resources to the intact field. Restor. Neurol. Neurosci..

[56] Lijffijt M (2009). P50, N100, and P200 sensory gating: relationships with behavioral inhibition, attention, and working memory. Psychophysiology.

[57] Kleinhans NM (2011). NeuroImage fMRI evidence of neural abnormalities in the subcortical face processing system in ASD. Neuroimage.

[58] Belmonte M (2000). Abnormal attention in autism shown by steady-state visual evoked potentials. Autism Int. J. Res. Pract..

[59] Hughes JR (2009). Update on autism: a review of 1300 reports published in 2008. Epilepsy Behav..

[60] Villalobos ME, Mizuno A, Dahl BC, Kemmotsu N, Müller R-A (2005). Reduced functional connectivity between V1 and inferior frontal cortex associated with visuomotor performance in autism. Neuroimage.

[61] Tomasi D, Volkow ND (2019). Reduced local and increased long-range functional connectivity of the thalamus in autism spectrum disorder. Cereb. Cortex.

[62] Belmonte MK (2004). Autism and abnormal development of brain connectivity. J. Neurosci..

[63] Kleinhans NM (2008). Abnormal functional connectivity in autism spectrum disorders during face processing. Brain.

[64] Beker S, Foxe JJ, Molholm S (2018). Neuroscience and Biobehavioral Reviews Ripe for solution: delayed development of multisensory processing in autism and its remediation. Neurosci. Biobehav. Rev..

[65] Martínez-Sanchis S (2014). Neurobiological foundations of multisensory integration in people with autism spectrum disorders: the role of the medial prefrontal cortex. Front. Hum. Neurosci..

[66] Constantino J, Gruber C (2005). Social Responsiveness Scale (SRS): Manual.

[67] Döpfner M., Görtz-Dorten A., Lehmkuhl G., Breuer D., G. H. *Diagnostik-System für psychische Störungen nach ICD-10 und DSM-IV für Kinder und Jugendliche – II, DISYPS-II*. (Huber, Edison, 2008).

[68] World Health Organisation. *International Statistical Classification of Diseases and Related Health Problems, 10th Revision (ICD-10).* (WHO, 1992).

[69] Rühl, D., Bölte, S., Feineis-Matthews, S. & Poustka, F. *Diagnostische Beobachtungsskala für Autistische Störungen (ADOS)*. (Huber, 2004). 10.1024/1422-4917.32.1.45 14992047

[70] Bölte, S., Rühl, D., Schmötzer, G., & Poustka, F. *Diagnostisches Interview für Autismus – Revidiert. Deutsche Fassung des Autism Diagnostic Interview—Revised von Michael Rutter, Ann Le Couteur und Catherine Lord*. (Huber, 2005).

[71] Poustka, L., Rühl, D., Feineis-Matthews, S., Bölte, S., Poustka, F., & Hartung, M. *Diagnostische Beobachtungsskala für Autistische Störungen – 2*. (Huber, 2015).

[72] Weiß, H. R. & Weiß, B. *CFT 20-R. Grundintelligenztest Skala 2 – Revision*. (Hogrefe, 2006).

[73] Stefanou, M. E. Deficits in Social Cognition in Autism Spectrum Disorders and their Electro-Cortical Correlates : A Multisensory Integration perspective. (Albert-Ludwigs-Universität Freiburg, 2019).

[74] Brainard DH (1997). The psychophysics toolbox. Spat. Vis..

[75] Pelli DG (1997). The VideoToolbox software for visual psychophysics: transforming numbers into movies. Spat. Vis..

[76] Kleiner M, Brainard DH, Pelli DG (2007). What’s new in Psychtoobox-3?. Perception.

[77] Gondan M, Minakata K (2016). A tutorial on testing the race model inequality. Atten. Percept. Psychophys..

[78] American Electroencephalographic Society (1991). Guidelines for standard electrode position nomenclature. J. Clin. Neurophysiol..

[79] Ulrich R, Miller J, Schröter H (2007). Testing the race model inequality: An algorithm and computer programs. Behav. Res. Methods.

[80] Luck SJ (2005). An Introduction to the Event-Related Potential Technique.

[81] Maris E (2012). Statistical testing in electrophysiological studies. Psychophysiology.

[82] Maris E, Oostenveld R (2007). Nonparametric statistical testing of EEG- and MEG-data. J. Neurosci. Methods.

